# The Situation

**Published:** 1877-04

**Authors:** 


### EDITORIAL.

### The Situation.

The medical situation we mean, not the political, financial or commercial situation, but as we have numerous inquirers upon the medical situation in Buffalo and vicinity, we deem it not uninteresting to quite a number of young men just entering the arena of medical practice to hear something of the prospects offered here to the well-informed, honest, earnest workers, in our "glorious" profession.

Buffalo contains about 150,000 people liable to require the services of a physician; 20,000 of these are now supported by the poor department, and are really not very paying clients, though it must be confessed that it offers quite a large field for labor, valuable to the young man for experience, but not productive of large pecuniary returns ; 20,000 or 30,000 more of our population, if sick or disabled, are cared for by hospitals, asylums, dispensaries and other so-called charitable institutions not returning to physicians any consideration for their services.

This leaves us about 100,000 people open to require our services and expecting to be *charged* the usual fees for attendance. To supply this great want we have, as near as can be estimated, 216 physicians and at least twenty or thirty midwives, to whom may fairly be assigned the care in natural labor of nearly the entire German population. This divided equally will give one physician to every 420 people, who are to be treated by physicians, if sick, and *charged* the usual prices, not more than 50 per cent. of which can be reckoned upon as good. Average attendance annually has been estimated at one dollar each, which would afford, if equally divided, an annual salary of $420 *charged*, one-half off ; but it is not to be forgotten that some old and well established physicians attend ten times their proportion, and this mostly from the paying classes, thus lessening the chances to present aspirants for popular favor to a very great extent.

Comment upon all this is quite unnecessary, but says our correspondent now located in a neighboring village, "I have a large and tiresome ride, and if Buffalo is so well supplied with physicians who are so poorly paid, how is it that so many live in elegance and "fare sumptuously every day?" True, we have among us some who are well known in all Western New York and who are receiving the patronage of a wide circle of patrons; they scarcely draw their supplies from the city at all; are wholly independent of popular favor by reason of long lives of popular patronage, combined with business talent which few possess.

These are the men *you* can *see.* They have houses, and carriages and servants, and are always to be seen or heard of; but oftentimes their capable and intelligent co-laborers, who have not yet attained to their distinctions, cannot be seen or heard of even by common observers. However, *we* can see them in the future with perfect distinctness ; can see how they may rise to honor and fame ; how they may meet with true professional success ; but we cannot see how they are to obtain at such fearful odds the one all-absorbing, all-essential, everywhere coveted success of wealth or competence. Physicians who aim at riches should avoid Buffalo. Buffalo physicians are contented to strive to lay up treasure " where moth and rust doth not corrupt," and have long since abandoned the pursuit of earthly treasure. The field is open to all, and we most cordially invite good runners to join in the chase, promising good company, true, hearty, good fellowship, a jolly good time and a glorious reward.

